# On-chip *in vitro cell-network* pre-clinical cardiac toxicity using spatiotemporal human cardiomyocyte measurement on a chip

**DOI:** 10.1038/srep04670

**Published:** 2014-04-22

**Authors:** Tomoyuki Kaneko, Fumimasa Nomura, Tomoyo Hamada, Yasuyuki Abe, Hideo Takamori, Tomoko Sakakura, Kiyoshi Takasuna, Atsushi Sanbuissho, Johan Hyllner, Peter Sartipy, Kenji Yasuda

**Affiliations:** 1 Department of Biomedical Information, Division of Biosystems, Institute of Biomaterials and Bioengineering, Tokyo Medical and Dental University, Chiyoda, Tokyo 101-0062, Japan; 2 Medicinal Safety Research Laboratories, Kasai R&D Center, Daiichi-Sankyo Co. Ltd., Edogawa, Tokyo 134-8630, Japan; 3 Division of Biotechnology, IFM, Linköping University, SE- 581 83 Linköping, Sweden; 4 Cellectis Stem Cells, Cellartis AB, Arvid Wallgrens Backe 20, SE-413 46 Göteborg, Sweden; 5 These authors contributed equally to this work.; 6 Current address: Department of Frontier Bioscience, Hosei Univ., Koganei, Tokyo 184-8584, Japan.

## Abstract

To overcome the limitations and misjudgments of conventional prediction of arrhythmic cardiotoxicity, we have developed an on-chip *in vitro* predictive cardiotoxicity assay using cardiomyocytes derived from human stem cells employing a constructive spatiotemporal two step measurement of fluctuation (short-term variability; STV) of cell's repolarization and cell-to-cell conduction time, representing two origins of lethal arrhythmia. Temporal STV of field potential duration (FPD) showed a potential to predict the risks of lethal arrhythmia originated from repolarization dispersion for false negative compounds, which was not correctly predicted by conventional measurements using animal cells, even for non-QT prolonging clinical positive compounds. Spatial STV of conduction time delay also unveiled the proarrhythmic risk of asynchronous propagation in cell networks, whose risk cannot be correctly predicted by single-cell-based measurements, indicating the importance of the spatiotemporal fluctuation viewpoint of *in vitro* cell networks for precise prediction of lethal arrhythmia reaching clinical assessment such as thorough QT assay.

Lethal arrhythmia is one of the major safety concerns for developing drug candidate compounds^1^. The current integrated assay systems using *in vitro* assays such as hERG assay^2^ and isolated animal tissues (APD assay), and *in vivo* conscious and/or anesthetized whole animals (QT or MAP assay) still cannot fully predict the potential lethal arrhythmia including TdP or VT of drug candidates^3^, especially for false-negative^4^,^5^/positive^6^,^7^,^8^ compounds.

Emergence of human embryonic stem (hES) cells and human induced pluripotent stem (hiPS) cells provides the opportunity to access human cardiomyocytes (hCMs) for the early stage of cardiotoxicity as *in vitro* screening of human relevant cells before clinical testing^9^,^10^,^11^. The implementation of hCMs raises, however, the following two questions for global cardiac safety; whether replacement of animal cells with hCMs in the conventional *in vitro* screenings can give us more precise and accurate prediction of lethal arrhythmia in human; and, secondly, whether more precise complicated ventricular responses, such as TdP and/or VT/Vf can be evaluated using hCMs with newly developed *in vitro* assays, e.g., a new approaches of spatiotemporal measurement using an artificially constructed tissue-like hCM network model. As these two questions are fundamental and deeply related to the origin of the mechanism of cardiovascular arrhythmia, the answers will lead us to the establishment of more precise *in vitro* assay, including spatiotemporal aspects, so called ‘quasi-*in vivo* assay' using hCMs.

Lethal arrhythmia is caused by the increase of response uncertainty of single cardiomyocytes (temporal aspect)^12^ as a triggering factor and of cell-to-cell conductivity (spatial aspect) as an enhancement/suppression factor (Sup. Fig. 1)^13^,^14^. Increase of uncertainty of electrophysiological response of single cells could be the principal and essential origin of lethal arrhythmia triggering, and hence, the quantitative evaluation of fluctuation potential of single cells is critical and should be the first index for prediction of lethal arrhythmia. However, as the cells in the tissue present a functionally slightly different heterogeneity of responses, even under the same circumstances, we should consider the spatial viewpoint of cardiomyocytes because the lethal arrhythmia occurs in a tissue, i.e., cell community, not in single cells. Cell-to-cell conduction is also important to evaluate asynchronized signal propagation in the cardiomyocyte network in heart tissues. Those community effects of cardiomyocytes could be the second origin of arrhythmia in addition to the first origin, temporal fluctuation of repolarization time of single cells after depolarization. The second origin could have either enhancing or suppressing roles for lethal arrhythmia occurrence. For example, the heterogeneity of cardiomyocyte functional characteristics has a potential to enhance the occurrence of lethal arrhythmia because of the different responses of neighboring cells from the first origin. In contrast, the community effect of cardiomyocytes has also a potential to suppress the occurrence of lethal arrhythmia by the enhancement of synchronization tendencies with suppression of fluctuation in cell groups (Sup. Fig. 2)^15^,^16^. Hence, one promising way to predict ventricular arrhythmia at the *in vitro* level, is to measure the first origin of arrhythmia, i.e., temporal fluctuation of repolarization time of single cells, and then, measure the second origin, i.e., spatial fluctuation of conductivity of neighboring cardiomyocytes using spatially arranged hCMs^17^.

Spatial regulation of community size, cell network shape and orientation are the important features in the mechanism of higher complexity of cellular system like tissue and organ to resolve the limitations of conventional *in vitro* assay into the *quasi-in vivo* cell network assay^18^. For example, the community size of a cardiomyocyte cluster is important for the maintenance of stable beating intervals (Sup. Fig. 2), and the difference of community size and spatial network pattern also gives us different results using the same compounds^19^,^20^. Hence, as isolated single cells are not desirable for stable screening, we adopted hCM clusters as a model for temporal fluctuation measurement, and the following lined-up cell network assay as a model for spatial fluctuation measurement.

## Results

**Figure 1:**
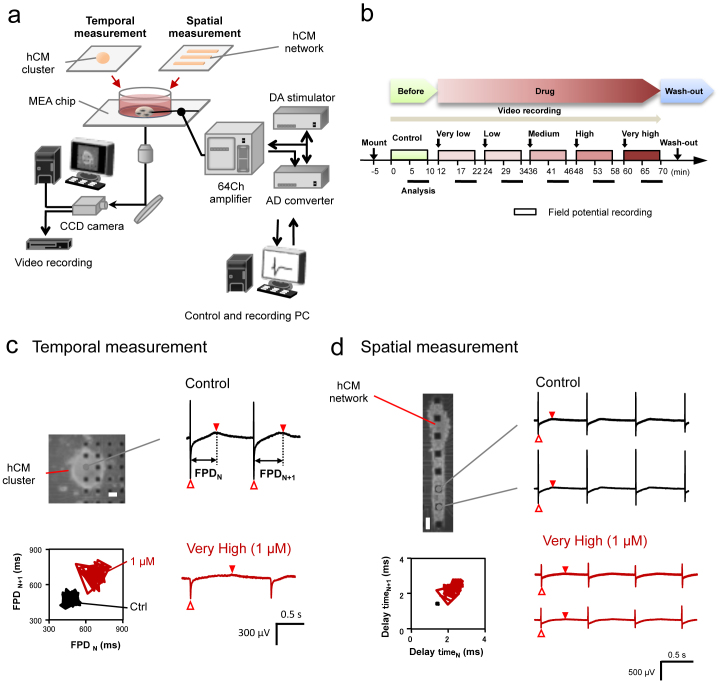
On-chip cell-network cardiotoxicity measurement using human cardiomyocytes. (a), Schematic diagram of on-chip cell-network multielectrode array (MEA) system for spatiotemporal functional measurement of cardiomyocyte networks using human cardiomyocyte (hCM) clusters for temporal aspect and rectangle hCM lined-up networks for spatial aspect (Fig. 1a is original and was drawn by K.Y., F.N., and T.H.). (b), The experimental protocol was designed for five-step increase of concentrations doses in each compound. Before recording, the incubated hCMs on the MEA chip was set in the system and placed for 5 min. Each step of recordings was for 10 min started from just after the compound application, and the last 5 min of the recording was adopted for analysis. After the experiments, the medium in the chip was exchanged into fresh medium for washing. (c), (d), field potential (FP) waveforms obtained from the hCM cluster for temporal measurement of FP duration (FPD) fluctuation (c), and from the lined-up hCM network for spatial measurement (d) in presence of compound (E-4031). Each phase contrast image was shown the hCM cluster (c) and the lined-up hCM network having same amount of cardiomyocytes (d) used for the experiments. Bars, 100 μm. The represented FP waveforms of 1 μM (the fifth- top dose very high concentration in (b)) were compared to those of control. The FPD for (c) was the time between the first inward sodium peak (open triangle) and second outward peak (closed reverse triangle). The conduction time for (d) was calculated from the propagation time of sodium inward peak (open triangle) of FP waveforms of cells on the neighboring electrodes. The fluctuations of the FPD and the conduction (delay) time after the addition of 1 μM E-4031 were shown in the lower graphs as Poincaré plottings.

To study the spatiotemporal increase of uncertainty (fluctuation) of hCM response, we have developed the on-chip cell network cultivation system, in which extra-cellular signals (field potentials: FP) of hCMs can be measured using a multi electrode array (MEA), and spatial arrangement control of cells can be performed using agarose microstructures designed on MEA chip (Fig. 1a, and Sup. Fig. 3). The drugs were applied to the medium in the MEA chip at 1% dilution in serially 5 step increasing-additions, and the FPs were measured for 10 min at each concentration (Fig. 1b). For the temporal fluctuation measurement, recorded FP duration (FPD) was defined as the time interval between the initial field-potential deflection and the peak of depolarization (Filled arrowheads in Figs. 1c, 1d; Sup. Fig. 1b and Sup. Fig. 4 also explains FPD intervals). The short-term variability (STV) of FPD defined as the mean distance of points perpendicular to the line of identity in the Poincaré plot, was calculated (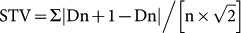, where Dn represents the FPD of n-th beating) (Graphs in Figs. 1c, 1d, and Sup. Fig. 1c)^21^. (For further details, see Supplementary Information.)

**Figure 2:**
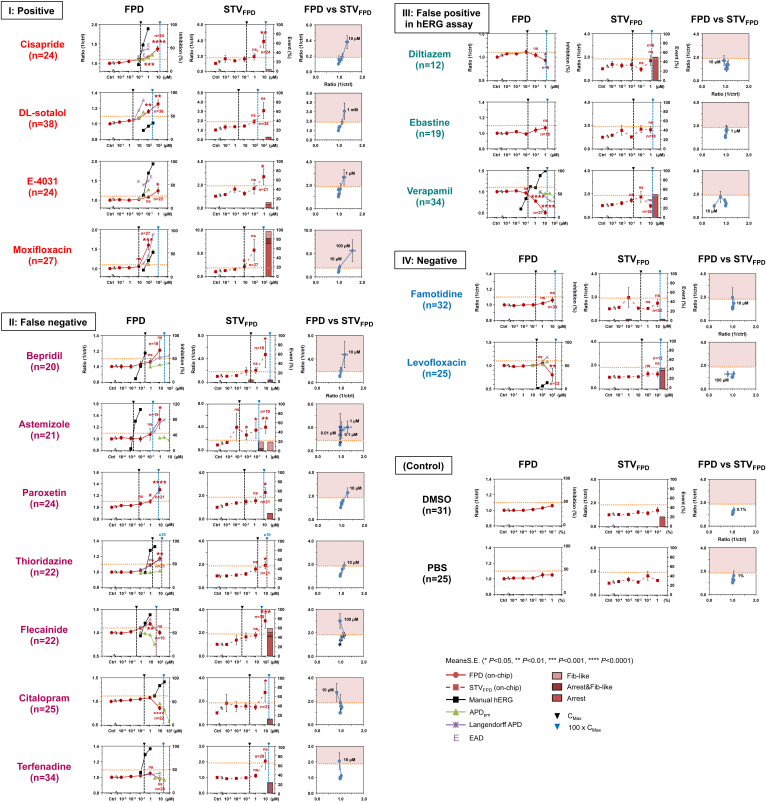
The effects of cardiotoxic compounds on FPD and STV_FPD_ of hCM clusters. I: Positive (Category I). II: False negative (Category II), III: False positive (Category III), IV: Negative (Category IV), and control. The compound concentration dependences on FPD (red circle), APD_90_ in papillary muscle (green triangle), APD_90_ in Langendorff hearts (purple cross), inhibition of hERG channel (black square) in left graphs. The compound concentration on STV_FPD_ (red square and dashed line) with each ratio of lethally abnormal events (Arrest, Arrest and fib-like, and Fib-like) in center graphs. The relationship between FPD and STV_FPD_ in the right graphs. The more than 1.9-fold of STV_FPD_ (pink) show a border as a high risk of VT/TdP.

First, we examined the temporal FPD prolongation in hCM clusters (CMCs), and compared with conventional methods such as *in vitro* hERG assay in hERG transfected CHO cells, APD assay in guinea-pig papillary muscle, *ex* *vivo* MAPD assay in rabbit Langendorff hearts with 16 representative compounds of four categories (see Table 1, and Fig. 2); positive (category I; positive in hERG assay, in APD assay and in clinical VT/TdP), false negative (category II; positive in hERG aasay, negative in APD assay, and positive in clinical VT/TdP), false positive in hERG assay (category III; positive in hERG assay and negative in both APD assay or clinical VT/TdP), negative (category IV; negative in hERG assay, APD assay, or in clinical VT/TdP) and control (phosphate-buffered saline (PBS), dimethyl sulfoxide (DMSO)).

The left graphs in Fig. 2 show the compound concentration dependence of FPD prolongation ratio (mean values of FPDs in a 5 min recording), hERG inhibition percent, APD prolongation ratio of papillary muscles, and MAPD prolongation ratio of Langendorff hearts in the 16 compounds (Table 1). For the interpretation of the FPD data, we set 10% prolongation of FPD as risk judgment, which met to the value of prolongation, 10%, in conventional APD measurements^22^ (see Sup. Fig. 4 for correlation of APD and FPD). Among the positive compounds (category I), FPD prolongation, APD prolongations of papillary muscles (APD_pm_) and MAPD of Langendorff hearts (MAPD_Lh_) showed the almost similar ability to detect QT risk (prolongation), which were consistent to the clinical results, whereas hERG inhibition ratio was not always representing the clinical results (e.g., DL-sotalol). Next, among the false negative compounds (category II), some of QT risk of the APD_pm_ negative compounds (astemizole, bepridil, paroxetine, and thioridazine) were correctly predicted by FPD prolongation measurement. However, FPD measurement was less predictive on the remaining compounds than the hERG assay, and MAPD_Lh_ (flecainide, terfenadine, and citalopram). Third, among the false positive compounds (category III), FPDs were consistent with the clinical results. Finally, among the negative control compounds (category IV), FPD prolongations were not observed.

The results indicate that the FPD prolongation measurement of hCMCs can predict the lethal arrhythmia risks in these four categories with a precision better than APD_pm_ but less than MAPD_Lh_. Hence, the hCMC FPD measurement could replace APD_pm_ measurement while maintaining the same quality of prediction. However, no obvious advantage of this replacement was observed since no perfect improvement of false negative/positive problems was measured. In addition, the result of famotidine in the FPD measurement raised concerns related to the advantage of FPD measurement against APD_pm_. From this viewpoint, as an answer to first question, “whether hCMs themselves can improve the predictive cardiotoxicity even using conventional measurement methods?”, we concluded that the replacement of animal CMs with hCMs in conventional *in vitro* APD measurement (i.e., FPD) assay did not give any substantially improvement for more precise predictive measurements.

That is, we need to expand the measurement method itself, not only replacing the cells but also the addition of better predictors of lethal arrhythmia, which can be used especially for these false negative compounds. Hence, we added the second index on the first index of averaged values of APD/FPD prolongations; that is, temporal fluctuation of hCM responses, because one of the origins of VT occurrence is uncertainty of cell responses such as fluctuation of repolarization time after depolarization.

To quantitatively evaluate the temporal fluctuation of hCM responses (fluctuation of FPD time in a series of beating), we adopted STV of FPD (STV_FPD_). STV is a well-known indicator of TdP occurrence in the electrocardiogram (ECG)^21^,^23^. Here we compared the temporally neighboring FP waveforms and FPD timings of identical cells, and evaluated these quantitatively for predicting the value of asynchronization.

**Figure 3:**
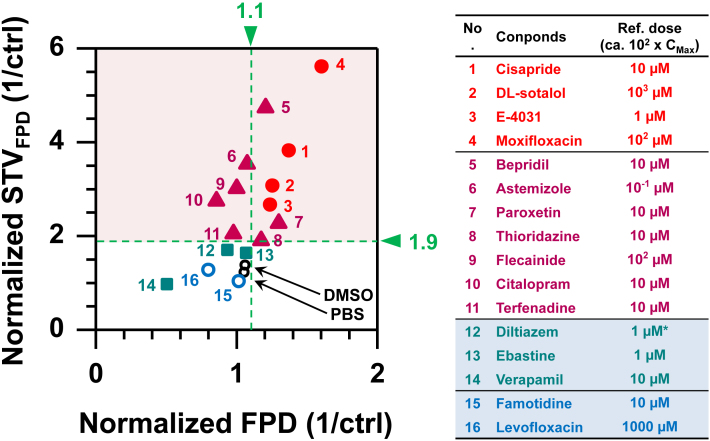
The summary of the effects of 10^2^ × C_MAX_ of cardiotoxic compounds on FPD and STV_FPD_ of hCM clusters. The normalized FPD and normalized STV_FPD_ of compounds at about 100 times of their effective therapeutic plasma concentration (~10^2^ × C_MAX_) were plotted. All the normalized STV values of positive and false negative compounds were higher than 1.9-fold, and all those of false positive and negative compounds were lower than 1.9 fold. In contrast, the normalized FPD values of false negative compounds spread both higher and lower of 1.1-fold.

In Fig. 2, the center graphs show the drug concentration dependence of STV_FPD_ changes in the above four compound categories. Using the standard positive compound E-4031, we defined that the border of risk in STV_FPD_ was 1.9, i.e., when STV increase over 1.9 from the STV_FPD_ results of the control, we consider that VT risk appeared. It should be noted that, in this case, as the value was experimentally defined using a particular source of hCMs (Cellartis AB), the risk value 1.9 might, to some extent, be dependent on the cell source. The right graphs in Fig. 2 indicate the relationship of FPD prolongation and STV_FPD_ increase. As shown for the compounds in the categories I, and II, FPD and STV_FPD_ correlated linearly for QT prolonging compounds. Moreover, STV_FPD_ measurement predicted the risk of the compounds in the category II, which FPD measurement couldn't predict their risks, except for terfenadine. Figure 3 also shows the summary of the FPD and STV_FPD_ in the above four compound categories at ca. 10^2^ times of effective therapeutic plasma concentration in consideration of the solubility. As shown in the graph, All the normalized STV_FPD_ of positive and false negative compounds were higher than 1.9 times, whereas those of false positive and negative compounds were lower than 1.9 times. In contrast, the tendency of normalized FPD of false negative was not consistent, and varied both higher and lower than 1.1 times.

**Figure 4:**
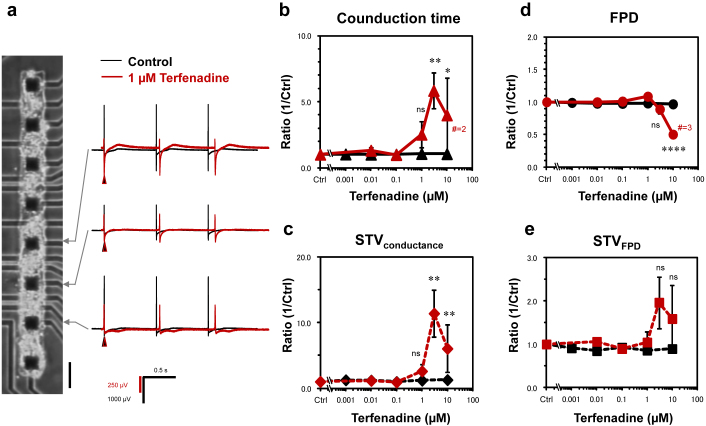
The spatial fluctuation measurement of terfenadine on the lined-up hCMs in on-chip quasi-*in vivo* measurement assay. The representative FP waveforms in the absence and the presence of 1 μM terfenadine (a). The phase contrast image was shown the lined-up hCMs used for the experiment. Bar: 100 μm. The conduction time (ms) was calculated from sodium inward peak (black triangle) of FP waveforms of neighboring electrodes. The concentration dependences of the conduction time and STV_conductance_ (the spatial fluctuation) in terfenadine were shown in (b) and (c). The concentration dependences of the FPD and STV_FPD_ (the temporal fluctuation) in terfenadine were shown in (d) and (e).

Terfenadine is well known as the only in hERG positive, and in clinical screening positive compounds. Although terfenadine has a strong affinity to the hERG channel, its strong inhibition of sodium ion channel might offset the QT prolongations due to hERG inhibition^24^,^25^,^26^. Even using conventional *in vitro* APD, hCM FPD and STV_FPD_ measurements, the clinical QT risk of terfenadine was hard to be predicted at the *in vitro* level from the viewpoint of temporal aspects of single cells. It might indicate the limit of the ability of temporal prolongation-based prediction and temporal fluctuation-based prediction. Hence, we have examined the potential of evaluation in spatial conduction time of cardiomyocyte network for terfenadine. In the spatial measurement assay, dispersed hCMs were applied into the rectangular shaped-agarose microchambers to form the linearly connected hCM networks having rectangular-shaped layer structures to maintain their community effects, and the number of cells in the network, ca. 1 × 10^3^, was in the same range as in hCM clusters used for temporal measurement (Sup. Fig. 3, and Table 1). The fluctuation of conduction time in the hCM network was measured by comparing FP waveforms of neighboring electrodes of a series of lined-up microelectrodes settled under the agarose microchambers. The peak of the sodium inward current was used for the indices of the conduction time of cell-to-cell conductance in hCM network (Fig. 1d, Fig. 4a and Sup. Fig. 1b), and the short-term variability (STV_Conductance_) of cell-to-cell conduction time defined as the mean distance of points perpendicular to the line of identity in the Poincaré plot, was calculated (Sup. Fig. 1c).

Figures 4b and 4c show the results of conductance propagation change (conduction time change) and of STV_Conductance_ caused by terfenadine application. As shown in the graphs, both the conduction time and the STV_Conductance_ increased depending on the increase of terfenadine concentration in the same manner and tendency. Hence, the clinical risk of terfenadine can also be predicted using the STV_Conductance_ as the fluctuation in spatial conductance viewpoint.

## Discussion

When cardiovascular safety is of concern after hERG assay, the next follow-up methods for interrogating for risk of TdP prior to animal studies have been lower throughput, *ex vivo* methods such as papillary muscle, Langendorff, Purkinje fiber, and ventricular wedge preparations^27^. As one of the potential candidates of a higher throughput approach that goes beyond hERG-mediated QT prolongation measurement, changes in FP waveforms in MEA measurements have been examined as a surrogate means to measure arrhythmias equal to the existing *ex vivo* measurements^11^,^12^,^28^,^29^. The results in our study indicated the limitation of the FPD measurement solely, and its ability showed the range of predictive accuracy of QT risk between APD_pm_ and MAPD_Lh_. However, we also have illustrated that the additional STV measurement of the temporal aspect (STV_FPD_) can predict the TdP risks of compounds, which were not detectable in the conventional *in vitro* prolongation-based measurements. Furthermore, STV measurement of the spatial aspect (STV_Conductance_) demonstrated the potential of more robust prediction of QT and/or lethal arrhythmic risks, which was not detected by the FPD and STV_FPD_ measurement, for compounds such as terfenadine.

A recent study reported the potential of correct prediction of terfenadine using the interdigitated electrode arrays for impedance measurement of the hCM 2-D sheet^30^. They pointed out the necessity of long-term measurement for multichannel blockers like terfenadine because of its delayed arrhythmic responses caused by time-dependent affinities for different ion channels especially hERG-inhibiting properties of terfenadine contributing to the torsadogenic liability masked by terfenadine's sodium ion channel inhibition. To examine the contribution of incubation time of terfenadine, we also evaluated the FPD prolongation in hCM clusters and its STV_FPD_, and found that a gradual increase of FPD was observed 8 h after 0.3 μM terfenadine was applied, and reached the risk level at 24 h (Sup. Fig. 5), whereas no significant increase in STV_FPD_ was observed even at 72 h. In contrast, as shown in Fig. 4, the STV_Conductance_ increased within 5 min after terfenadine was applied. One possible explanation for this phenomenon is that the inhibition of sodium ion channels caused by terfenadine, which contributes to offer the FPD prolongation due to hERG inhibition, introduced the fluctuation increase in cell-to-cell conduction time because of the decrease in sodium ion current to contribute to cell-to-cell communication adding to the increase in fluctuation of repolarization of cells. This is consistent to the Lu's group's report, in which, they observed non-TdP-like VT/VF without prolongation of the QT interval, and suggested that slowing of conduction via blockade of **IN**a (like Class Ic flecainide) may constitute a more important risk for terfenadine-induced cardiac death^31^.

The potential advantages of our cell-network-based *in vitro* assay, which could be regarded as ‘quasi-*in vivo* assay', include: (1) using a set of standard hCMs prepared from human pluripotent stem cells of different races, sexes, and also from patients with various diseases to provide an ideal testing panel platform; (2) to predict lethal arrhythmia by evaluation of the temporal fluctuation of ion channels kinetics in cells, and by evaluation of spatial cell-to-cell conduction time fluctuation using the on-chip cell network having limited linear conductance pathways of hCMs in rectangular-shaped microchambers.

In conclusion, we have shown the potential of *in vitro* predictive measurement of cardiac arrhythmia using hCMs. The electrophysiological measurement of prolongation of FPDs with hCMs showed that (1) FPD prolongation measurement has the ability to detect QT risk and pro-arrhythmic risks almost similar to the conventional *in vitro* measurements such as APD_pm_ and MAPD_Lh_, without significant improvement of the false-positive/negative problems; (2) temporal fluctuations (STV_FPD_) measurement predicted ventricular arrhythmia risks more precisely in most of the representative compounds including false-positive/negative compounds except for terfenadine; (3) even the risk of those false-negative drugs such as terfenadine was predicted using the fluctuation measurement of spatial conduction of the cell-to-cell connections in the cardiomyocyte network. Thus, only applying hCMs for conventional *in vitro* screening is not enough to get more precise prediction of arrhythmia occurrence, whereas, the combination of hCMs with the new approach of spatiotemporal measurement of temporal STV_FPD_ and spatial STV_Conductance_ give us an potential of global predictive arrhythmic cardiotoxicity measurement beyond existent hERG and APD/QT assays as a *cell network* assay to reach to the *quasi in vivo* screening.

## Methods

### Drugs

The following 16 compounds in four categories were chosen and applied for experiments: Positive (category I; positive in ether-a-go-go-related gene (hERG) assay, action potential duration (APD) assay, and in clinical VT/TdP) four compounds were cisapride, DL-sotalol E-4031, and moxifloxacin; false negative (category II; positive in hERG assay, negative in APD assay, and positive in clinical VT/TdP) seven compounds were bepridil, astemizole, paroxetin, thioridazine, flecainide, citalopram, terfenadine; false positive in hERG assay (category III; positive in hERG assay, and negative in both ADP assay or clinical VT/TdP) three compounds were diltiazem, ebastine, verapamil; and negative (category IV; negative both in hERG assay and in clinical VT/TdP) two compounds and two control references were levofloxacin, famotidine, phosphate-buffered saline (PBS), and dimethyl sulfoxide (DMSO). The administration concentration of each measurement and suppliers of drugs are listed in Sup. Table 1. All drugs except for nicorandil, moxifloxacin and levofloxacin, were purchased from Sigma (St. Louis, MO). Nicorandil was purchased from Wako Chemical (Osaka). Moxifloxacin and levofloxacin were obtained from Daichi-Sankyo. Compound stocks were prepared in dimethyl sulfoxide (DMSO) or dH_2_O at 30 ~ 100 times of their effective therapeutic plasma concentration (C_eff_) in consideration of the solubility. Compound stocks were serially diluted in maintenance media in a separate 96-well tissue culture plate (Corning).

### Culture of human embryonic stem cell-derived cardiomyocyte cluster

All experiments were conducted with synchronously beating, confluent, non-dividing hES-CMC™ acquired from Cellartis, AB (www.cellartis.com). The cells were maintained in DMEM supplemented with 1 mM GlutaMAX, 100 U/mL penicillin, 0.1 mg/mL streptomycin, 1% nonessential amino acid, 0.1 mM β-mercaptoethanol, and 20% heat-inactivated fetal bovine serum (Invitrogen, Carlsbad, CA, USA). The hES-CMC™s were incubated at 37°C in 5% CO_2_ overnight after the delivery and transferred to the MEA chips. The clusters on the chips were incubated for 4 days prior to experiments. During culturing, the medium was replaced every 2–3 days.

### Field potential recordings using on-chip MEA system

Self-designed and -made MEA chips were prepared. First, the surface of chip was coated with collagen type I-C (Nitta Gelatin, Japan). Beating hCMCs were plated on the electrodes and incubated at 37°C in a humidified atmosphere of 95% air and 5% CO_2_. Extracellular potential recordings of the beating hCMCs were performed using the self-made on-chip MEA system at a sampling rate of 10 kHz with low path filter of 2 kHz and high path filter of 1 Hz, and amplified by 100–50,000 using the amplifier. All MEA measurements were performed at 37°C.

### Drug administration protocol

The clusters for drug assay were selected by their beating frequency (0.3–1.5 Hz) and the waveforms of field potential (FP) recordings (Sup. Fig. 6). The volume of the medium in the chip was measured before drug assay. First, the chip with clusters was placed in the holder of on-chip MEA system, and equilibrated for 5 min, and then the control FP waveforms were recorded for 10 min. Subsequently, the drug was applied to the medium at 1% dilution in serially increasing additions, and the FP waveforms were recorded for 10 min at each concentration. Finally, the medium was replaced with fresh medium after washing with the fresh medium three times. The last 5 min extracted from 10 min recorded FP waveform data was used for FP duration (FPD) measurement at each concentration. The FPD was defined as the duration time between the initial field-potential deflection and the peak of inward current of depolarization mainly caused by potassium ion channels. The FPD were normalized (cFPD) for beating rate of the cardiomyocytes using Bazett's correction formula. The short-term variability (STV) of FPD defined as the mean distance of points perpendicular to the line of identity in the Poincaré plot, was calculated (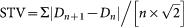, where D_n_ represents the FPD of n-th beating).

### Statistical analysis

All values are presented as mean ± S.E.M. (unless stated otherwise). Drug effects at high or very high concentration were performed using the unpaired Student's t-test. Dunnett's Multiple Comparison Tests were used when comparing multiple groups. *P* < 0.05 was considered as statistically significant.

## Supplementary Material

Supplementary Information Supplementary information

## Figures and Tables

**Table 1:** Comparison of compound safety assays (hERG assay, APD assay in papillary muscle, Langendorff assay and on-chip MEA assay) for predictive clinical VT/TdP risk

Category	Drug	Clinical report		*in vitro* assay				On-chip MEA system								
VT/VF/TdP	C_MAX_ (μM)	hERG inhibition (%)	APD_90_ prolongation in papillary musole (ratio)	MAPD_90_ prolongation in Langendorff hearts (ratio)	Ref. dose (10 × C_MAX_)	FPD (ratio)	*t*-test	STV_FPD_ (ratio)	*t*-test	Ref. dose (ca. 10^2^ × C_MAX_)	Arrest (%)	Fib-like (%)	n	Score		
I: Positive	Cisapride	(+)	0.18	90.6	1.14	1.37^E^	1 μM	1.37 ± 0.07	****	3.82 ± 0.85	**	10 μM	0	4	25	High
DL-sotalol	(+)	2.5	19.2	1.15	1.30	30 μM	1.25 ± 00.05	**	3.08 ± 0.86	ns	10^3^ μM*^7^	5	0	38	High	
E-4031	(+)		74.5	1.26	1.50^E^	0.1 μM	1.24 ± 0.08	*	2.67 ± 0.67	*	1 μM	8	4	24	High	
Moxifloxacin	(+)	10	51.4	1.44	1.87	300 μM	1.60 ± 0.12	***	5.61 ± 2.31	ns	10^2^ μM	0	0	27	High	
II: False negative on APD in papillay muscle	Bepridil	(+)	0.30	61.9*1	1.02	1.12	10 μM	1.21 ± 0.12	ns	4.74 ± 2.26	*	10 μM	5	5	20	High
Astemizole	(+)	0.002	105.4*2	1.01	1.27^E^	1 μM	1.08 ± 0.10	ns	3.54 ± 1.39	*	10^-1^ μM	0	14	25	High	
Paroxetin	(−/+)	0.07	(n.d.)	(n.d.)	(n.d.)	(n.d.)	1.30 ± 0.06	****	2.27 ± 0.41	*	10 μM*^7^	13	0	24	High	
Thioridazine	(+)	1.8	87.8*3	1.01	1.16	10 μM	1.17 ± 0.03	**	1.90 ± 0.30	*	10 μM	0	0	22	High	
Flecainide	(+)	0.43	88.3*4	0.75	1.29*6	30 μM	1.00 ± 0.05	ns	3.01 ± 0.65	***	10^2^ μM*^7^	13	0	24	High	
Citalopram	(+)	0.27	83.5	0.95	(n.d.)	10 μM	0.86 ± 0.04	****	2.75 ± 0.78	*	10 μM	12	0	25	High	
Terfenadine	(+)	0.22	94.2*5	0.98	1.05	10 μM	0.98 ± 0.04	ns	2.05 ± 0.53	ns	10 μM	21	3	34	High	
III: False positive on hERG	Diltiazem	(−)	0.11	(+)	(−)	(n.d.)	10 μM	0.93 ± 0.08	*	1.70 ± 0.43	ns	1 μM*^8^	50	0	12	Low
Ebastine	(−)	0.16	(+)	(−)	(−)	0.3 μM	1.07 ± 0.03	ns	1.64 ± 0.28	ns	1 μM	0	0	18	Low	
Verapamil	(−)	0.17	99.2	0.95	0.79	10 μM	0.51 ± 0.05	****	0.97 ± 0.18	ns	10 μM	49	0	43	Low	
IV: Negative	Famotidine	(−)	0.19	(−)	(−)	(−)	10 μM	1.02 ± 0.02	ns	1.03 ± 0.09	ns	10 μM	0	0	32	Low
Levofloxacin	(−)	22	13.2	1.07	1.18	300 μM	0.80 ± 0.14	**	1.27 ± 0.25	ns	10^3^ μM	40	4	25	Low	
DMSO	(−)		(n.d.)	(n.d.)	(n.d.)	(n.d.)	1.06 ± 0.02	-	1.38 ± 0.24	-	0.1%	6	0	31	No	
PBS	(−)		(n.d.)	(n.d.)	(n.d.)	(n.d.)	1.05 ± 0.02	-	1.23 ± 0.11	-	-	0	0	25	No	

Data on hERG inhibition, APD prolongation in papillary muscle and in Langendorff hearts, and on-chip MEA assay was based on our results (Figs. 2). + and – show the positive and negative risk on the results of our assays. Short show the APD/FPD shortening on the results of our assays. (+) or (−) in present the positive or negative risk based on the references and pharmaceutical attachments. The data (the relative ratio against the control) is shown as only mean for on hERG inhibition, APD prolongation in papillary muscle and in Langendorff hearts and as mean ± S.E. for on-chip MEA assay. The reference concentrations (Ref. dose) show the concentration referred the experiments (hERG assay, APD assay in papillary muscle, Langendorff assay). VT/TdP risk is compiled from the literature.

Eindicates EAD appearances on the Langendorff assays.

*1: 0.3 μM bepridil;

*2: 0.1 μM astemizole;

*3: 3 μM thioridazine;

*4, *^6^: 10 μM flecainide;

*5: 1 μM terfenadine.

Abbreviations: phosphate-buffered saline (PBS), and dimethyl sulfoxide (DMSO). Red hatched area indicates positive (+risk), and blue area indicates negative (-risk) judgment using each method.
