# Supplementary material for: On-chip in vitro cell-network pre-clinical cardiac toxicity using spatiotemporal human cardiomyocyte measurement on a chip
Source: Sci Rep. 2014 Apr 22;4:4670. doi: 10.1038/srep04670 (PMC5381194; doi:10.1038/srep04670)
Supplement: Supplementary Information [file srep04670-s1.pdf]

Supplementary Information [for online]

On-chip *in vitro cell-network* pre-clinical cardiac toxicity using spatiotemporal human cardiomyocyte measurement on a chip

Tomoyuki Kaneko[†], Fumimasa Nomura[†], Tomoyo Hamada[†], Yasuyuki Abe, Hideo Takamori, Tomoko Sakakura, Kiyoshi Takasuna, Atsushi Sanbuissho, Johan Hyllner, Peter Sartipy & Kenji Yasuda

Supplementary Explanation of Methods [in online]

Measurement of potassium (K^+) current by patch clamp in hERG expressing CHO-K1 cells

A patch clamp glass electrode was filled with internal solution (KCl: 130 mM, $MgCl_2$: 1 mM, EGTA: 5 mM, MgATP: 5 mM, HEPES: 10 mM, pH 7.20). The electrode was attached to a patch clamp headstage holder and the tip of the electrode was brought into contact with the cell membrane with a micromanipulator under the microscope. Slight negative pressure from suction was created inside the electrode and the patch clamp system AXOPATCH 200B (Axon Instruments) and Clampex 9.2 was used to obtain a measured membrane resistance value (R-memb) greater than 20 megohms. The resting membrane potential was set at -80 mV just to wait to obtain R-memb greater than 1 gigaohms. Then to make whole-cell clamp mode, a strong suction was applied to rupture the cell membrane under the electrode. Cell capacitance and series resistance were compensated. The stimulation protocol (holding potential: -80 mV, depolarization pulse: +50 mV, for 2 s, repolarization pulse: -40 mV, for 2 s, pulse frequency:

0.067 Hz) was applied and the corresponding current was recorded. After the current waveform became stable, the application solutions were added cumulatively from the lowest concentration. The cells were exposed to each concentration for 5 minutes and washed with Tyrode solution (NaCl: 137 mM, KCl: 5.4 mM, $CaCl_2$: 2 mM, HEPES: 10 mM, $MgCl_2$: 1 mM, Glucose: 10 mM, pH 7.40) to observe the recovery after the exposure to the highest concentration. All experiments (n=3) were carried out at room temperature. The cells were perfused with Tyrode solution at the rate of 2.5 ml/min through the experiment as the background flow. The test compound was added to the cells directly via a pipette set nearby the cells at the rate of 350-400 μl/min. Percentage change from pre-application values in tail peak current (% inhibition) was calculated using the following formula: % inhibition = [1-average of the 17th to 20th tail currents after application/average of the consecutive 4 currents just before the start of the first application (the lowest one)] × 100. The dose levels were selected to describe the dose response curve. The maximum concentration was set to at least approximately 30~100 times its effective therapeutic plasma concentration in consideration of the solubility.

Action potential recording in guinea pig right ventricular papillary muscle

Action potentials were recorded in ventricular papillary muscle preparations obtained from the right ventricles of 4-12 weeks Hartley male guinea pigs (Japan SLC, Hamamatsu Japan). Each preparation was immersed in Krebs-Henseleit buffer at 36.5 ± 0.5 °C aerated with a mixture of 95% O_2 and 5% CO_2, and stimulated using rectangular pulses (1 ms duration with a frequency of 1 Hz and an intensity about 1.3 times the diastolic threshold) provided by an electrical stimulator (SEN-3201, Nihon Koden, Tokyo, Japan) until the end of recording. Transmembrane action potentials were recorded using a 3 M KCl glass microelectrode with a tip resistance of 5 - 30

megohms, which was coupled to an Ag-AgCl bath electrode and connected to a microelectrode amplifier (MEZ-8300, Nihon Koden). Transmembrane action potentials were displayed on an oscilloscope (CS-4025, Kenwood, Tokyo, Japan), and recorded and analyzed by an external computer system (WinCAPA1.6.6.Aui, Physio-Tech). The components of the action potential analyzed by the computer system are: resting membrane potential (RMP), action potential amplitude (APA), overshoot (OS), action potential duration at 30%, 50% and 90% of repolarization (APD_{30}, APD_{50} and APD_{90}, respectively) and maximum rate of rising phase (V max = dV/dt max). After an equilibration period for at least one hour, the test compound was added to the organ bath at increasing cumulative concentrations. The administration volume and rate was 20, 40, 140 µl/20 ml organ bath and 1 s/dose. Cardiac action potentials were recorded before and 30 minutes after the exposure of the test compound at each concentration. The dose levels were selected to describe the dose response curve.

Monophasic action potential duration (MAPD) measurements in isolated Langendorff-perfused female rabbit heart

Female NZW rabbit were anesthetized by an intravenous injection of thiopental (30 mg/kg) and heparin (500 U/kg). Then the heart was quickly removed and its aorta was cannulated, the bundle of His was sectioned, for perfusion of the coronary artery according to the Langendorff method with constant pressure (initial perfusion pressure: 60-70 mmHg). The heart was perfused at 36.5± 0.5ºC and a flow rate of about > 24 ml/min with Krebs-Henseleit buffer (NaCl: 118 mM, KCl: 4.7 mM, $CaCl_2$: 2.5 mM, $MgSO_4$: 1.2 mM, KH_2PO_4: 1.2 mM, $NaHCO_3$: 25 mM, glucose: 11.1 mM) oxygenated with a mixture of 95% O_2 + 5% CO_2. Two electrocardiogram (ECG) electrodes were held lightly against the epicardium, the positive one on the left ventricle, the

negative one on the electro cannula. A monophasic action potential (MAP) recording electrode was attached to the wall of the left ventricle to obtain an epicardial MAP signal. A bipolar electrode was attached onto the ventricle for pacing (1 ms duration, 2 times threshold voltage, 1Hz) by an electronic stimulator (SEN-3201, Nihon Koden). The ECG and MAP were amplified with an RMP-6004M amplifier system (Nihon Koden) and a DAM50 system (World Precision Instruments, Sarasota, FL, USA), respectively. All signals were collected on a computer with software (WinVAS3, Physiotec, Tokyo, Japan). The heart was allowed to stabilize for ≥ 30 min after being mounted on the Langendorff apparatus and studies were commenced after stabilizing the ECG and MAP signals. We calculated MAPD measured at 90% repolarization (MAPD90). The cells were perfused with Tyrode solution at the rate of 2.5 ml/min through the experiment as the background flow. The test compound was added to the cells directly via a pipette set nearby the cells at the rate of 350-400 μl/min. The dose levels were selected to describe the dose response curve. The maximum concentration is set to 30~100 times its effective therapeutic plasma concentration in consideration of the solubility.

Supplementary Data [in online]

Supplementary Table 1. Reference data of compounds.

Category	Drug	Class/applications	Product name	Product number	Supplier	hERG assay (uM)	APD in papillary muscle (uM)	Langendorff (uM)	MEA system (uM)	Stock for MEA system
I: Positive	Moxifloxacin	Fluoroquinolone antibiotics	Moxifloxacin Hydrochloride	-	gift from Daiichi Sankyo Co., Ltd	30,100,300	100, 300	30,100,300	0.1, 1, 10, 100, 1000	100 mM in DMSO
	E-4031	Class III antiarrhythmic, hERG K+ channel blocker	E-4031	M5060	Sigma	0.03,0.1,0.3	0.01,0.03,0.1	0.03,0.1	0.0001, 0.001, 0.01, 0.1, 1	1 mM in DDW
	Cisapride	5-HT4 serotonin receptor agonist	Cisapride	C4740	Sigma	0.1,0.3,1	0.1,0.3,1,3	0.1,0.3,1	0.001, 0.01, 0.1, 1, 10	10 mM in DMSO
	D-sotalol	Class III antiarrhythmic, beta-adrenoceptor blocker	D-sotalol hydrochloride	-	gift from Dr. Sugiyama	-	-	-	0.1, 1, 10, 100, 1000	100 mM in DDW
	DL-sotalol		(±)-Sotalol hydrochloride	S0278	Sigma	30,100,300	10,30	3,10,30	0.1, 1, 10, 100, 1000	100 mM in DDW
II: APD false negative	Astemizole	H1 histamine receptor antagonist	Astemizole	A6424	Sigma	0.01,0.03,0.1	1,3,10	0.3,1,3	0.0001, 0.001, 0.01, 0.1, 1	1 mM in DMSO
	Bepridil	Class IV antiarrhythmic	Bepridil hydrochloride	B5016	Sigma	0.03,0.1,0.3	1,10,100	1,10,100	0.001, 0.01, 0.1, 1, 10	100 mM in DMSO
	Paroxetin	Antipsychotic	Paroxetine maleate	P1372	Sigma	-	-	-	0.001, 0.01, 0.1, 1, 10	10 mM in DMSO
	Thioridazine	Antipsychotic	Thioridazinehydrochloride	T9025	Sigma	0.3,1,3	0.1,1,10	0.1,1,10	0.001, 0.01, 0.1, 1, 10	10 mM in DDW
	Pimozide	Antipsychotic	Pimozide	P1793	Sigma	0.03,0.1,0.3	0.1,1,10	0.1,1,10	0.001, 0.01, 0.1, 1, 10	10 mM in DMSO
	Flecainide	Class IC antiarrhythmic, Na+ channel blocker	Flecainide acetate salt	F6777	Sigma	1,3,10	1,3,10,30	1,3,10	0.01, 0.1, 1, 10, 100	10 mM in DDW
	Citalopram	Antidepressant	Citalopram hydrobromide	C7861	Sigma	3,10,30	10,30,100	-	0.001, 0.01, 0.1, 1, 10	10 mM in DDW
	Nicorandil	K+ ATP channel activator	Nicorandil	65141-46-0	Wako	-	30,100,300	-	0.01, 0.1, 1, 10, 100	100 nM in DMSO
	Terfenadine	H1 histamine receptor antagonist	Terfenadine	T9652-5G	Sigma	0.1,0.3,1	3,10,30	0.1,1,10	0.001, 0.01, 0.1, 1, 10	10 mM in DMSO
III: Negative	Amiodarone	Class III antiarrhythmic	Amiodarone Hydrochloride	A8423	Sigma	0.1,0.3,1	-	-	0.001, 0.01, 0.1, 1, 10	100 mM in DMSO
	Ebastine	H1 histamine receptor antagonist	Ebastine	E9531	Sigma	-	-	-	0.0001, 0.001, 0.01, 0.1, 1	1 mM in DMSO
	Verapamil	L-type calcium channel blocker	Verapamil Hydrochloride	V4629	Sigma	0.03,0.1,0.3,1,3,10	1,3,10,30,100	1,10,30	0.001, 0.01, 0.1, 1, 10	10 mM in DDW
	Diltiazem	calcium channel blocker	Diltiazem Hydrochloride	-	gift from MCM	-	-	-	0.001, 0.01, 0.1, 1, 10	10 mM in DDW
	Famotidine	H2 histamine receptor antagonist	Famotidine	F6889	Sigma	-	-	-	0.01, 0.1, 1, 10, 100	100 mM in DMSO
	Levofloxacin	Fluoroquinolone antibiotics	Levofloxacin	-	gift from Daiichi Sankyo Co., Ltd	30,100,300	10,100,300	100,300	0.1, 1, 10, 100, 1000	100 mM in DMSO
Background	PBS	-	PBS(Phosphate Buffered Salts)Tablets	T900	Takara	-	-	-	0.0001, 0.001, 0.01, 0.1, 1%	-
	DMSO	-	Dimethyl sulfoxide	D8418	Sigma	-	-	-	0.00001, 0.0001, 0.001, 0.01, 0.1%	-

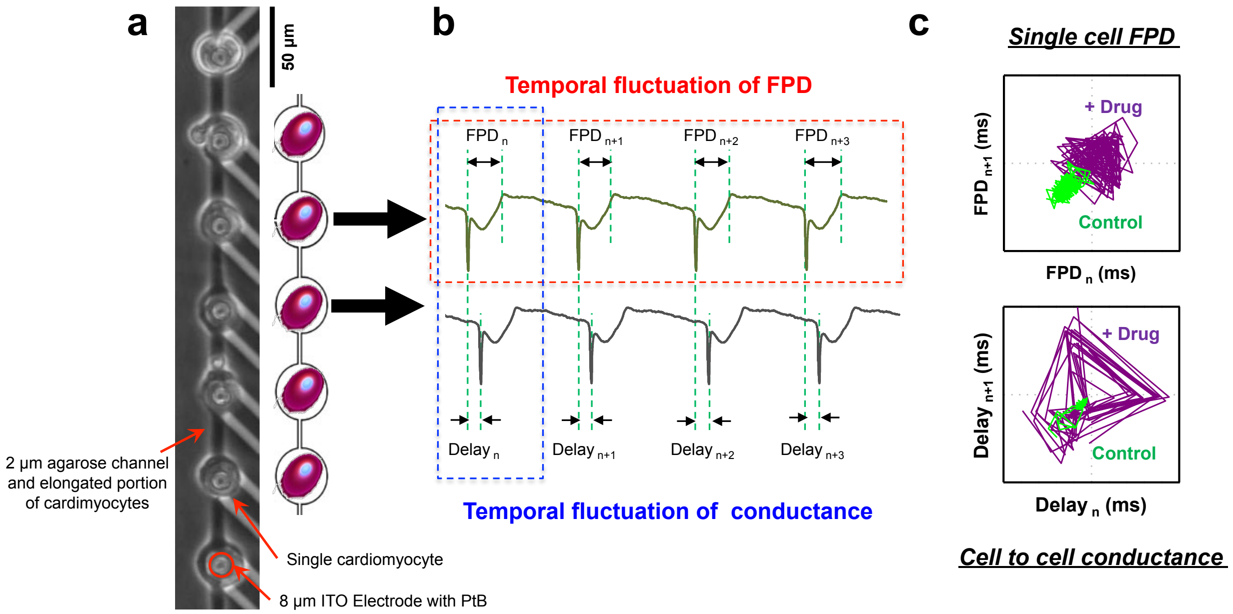

Supplementary Figure 1. Concept of single-cell-based on-chip re-entry model for pre-clinical testing for TdP prediction. The network formation enables us to make a model of the signal propagation in the heart tissue. **a**, Phase contras image of line-upped cell-network. Bar: 50 μm. **b**, Field potential recordings obtained from each single cell on single electrode. Temporal changes of field potential duration (FPD) between depolarization and repolarization peak and conduction time on cell to cell, or pacemaker cell to target cell conduction time are calculated. **c**, Poincaré plots of Single cell FPD (upper graph) and cell to cell conduction time (lower graph). In addition of TdP positive drug (purple lines), short-term fluctuation of FPD or conduction time are increased.

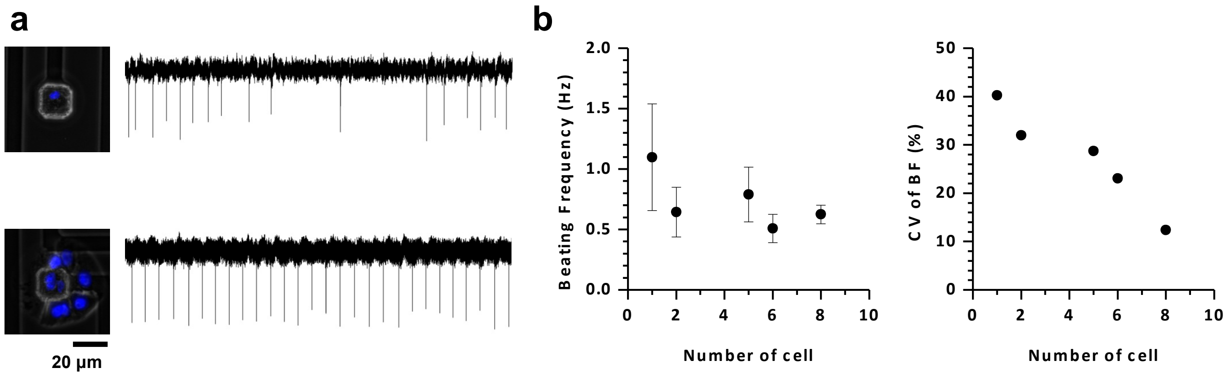

Supplementary Figure 2. Community effect of beating rhythm and waveform of field potential for beating rhythm stabilization. a, Beating rhythm of isolated single cell (upper image and trace) and 8 (lower image and trace) cell community cultivated on single electrode (day 3 after seeding on the MEA chips (3DIV)). Superimposed phase contrast and fluorescent images of cells and nuclei stained by Hoechst 33342 are indicated. Bar: 20 μm. **b**, Community size dependence of the beating frequency (Left graph, mean±SD) and their fluctuations (right graph) to the cell number in cell communities.

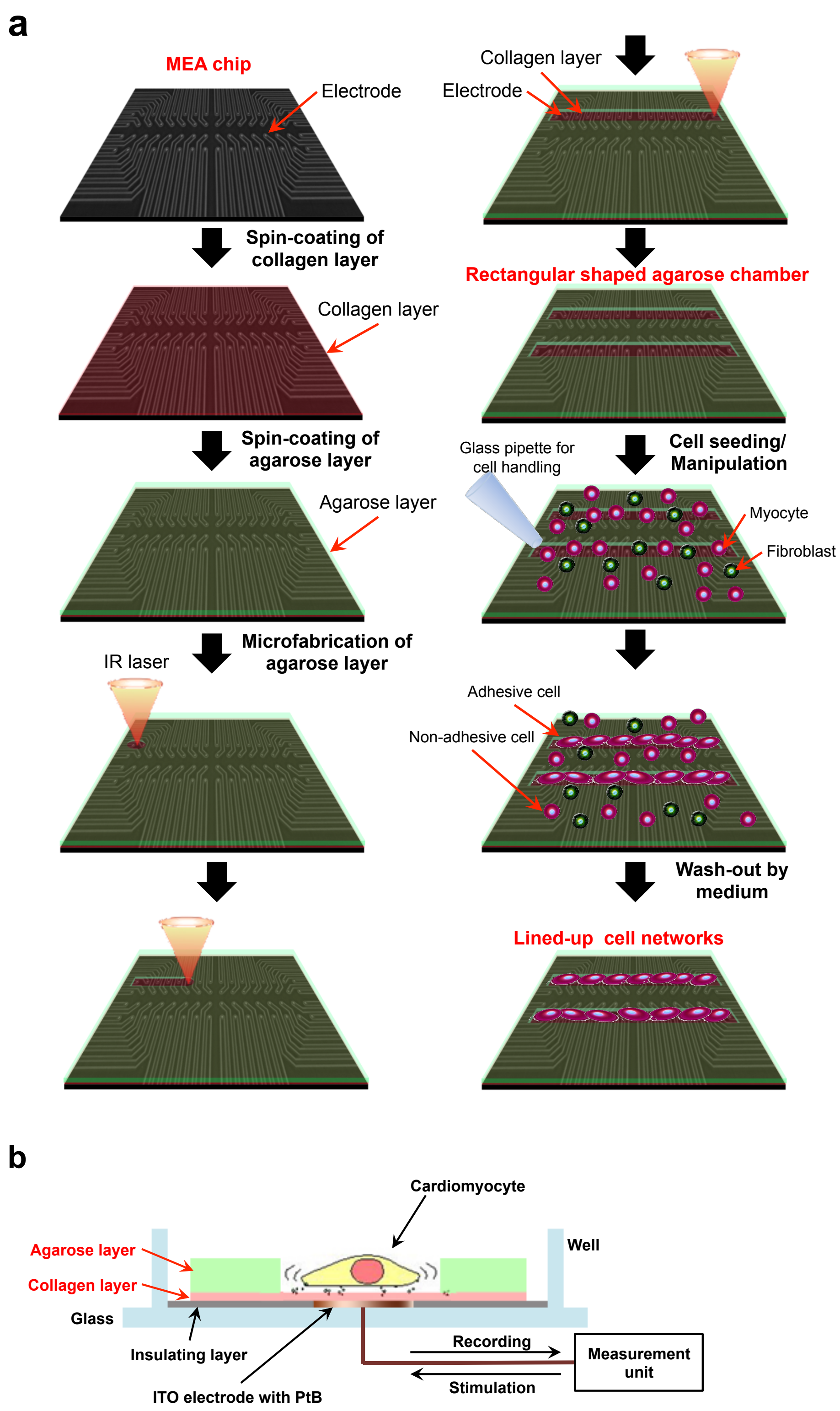

Supplementary Figure 3. Variety of beating properties on hES-CMs clusters. a, Fabrication procedure of lined-up cardiomyocyte network. **b**, Cross-sectional view of cell chip.

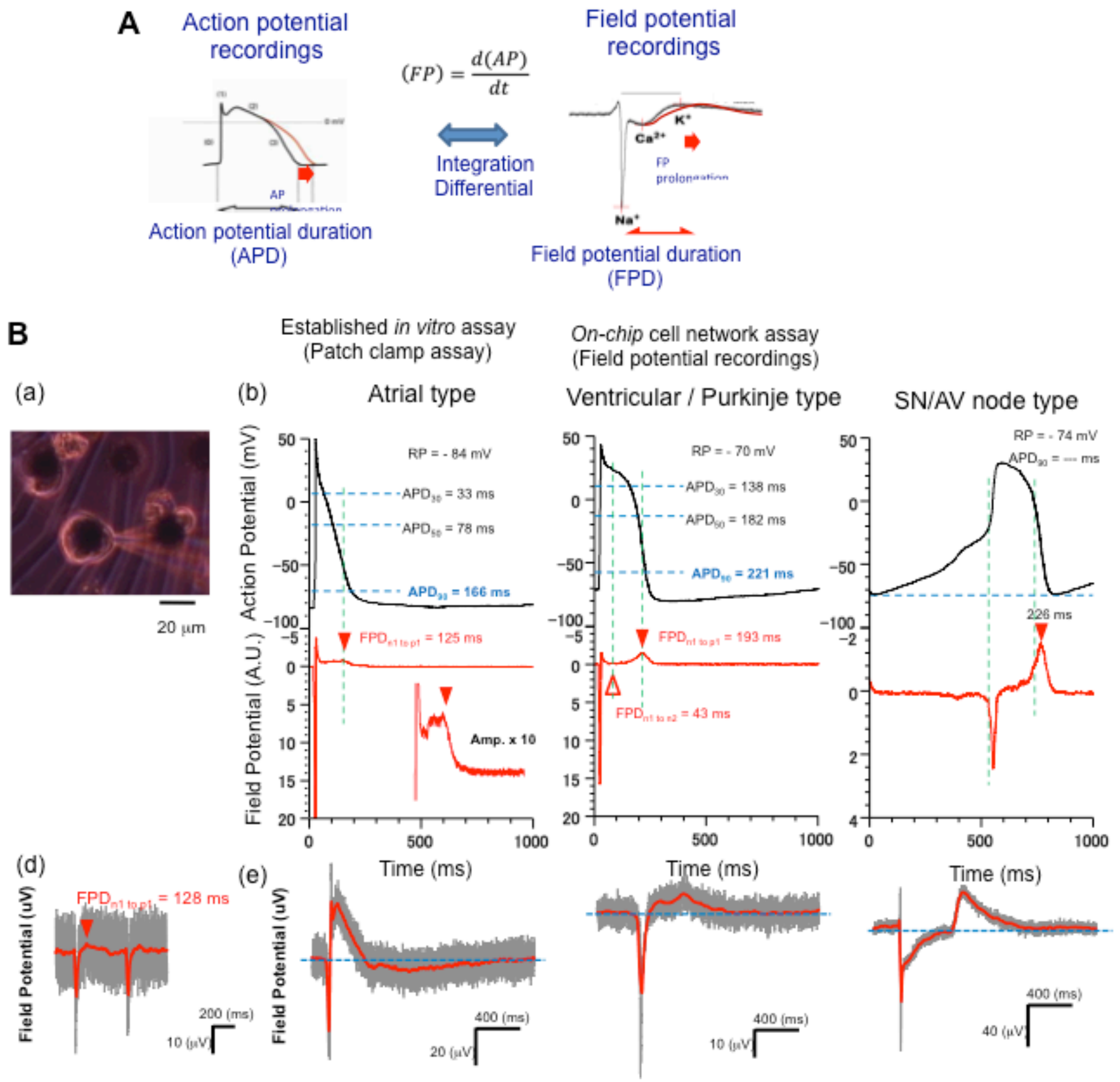

Supplementary Figure 4. Comparison of waveform and duration between action potential recordings (AP recordings) and its differential. Upper Schematic Images. A. A schematic drawing of relationship of action potential and field potential. **B.** (a) Phase contrast image of human ES derived cardiomyocytes under parch clamp assay. Bar: 10 um. (b) Waveform of AP recordings (upper traces) at 1 Hz pacing and its differential curves (lower traces) . Differential data were processed by smoothing at 20 point (4 ms). (c) Phase contrast image of single cells on microelectrode. Bar: 10 um. (d) Waveform of FP recordings from single cell with 2,2 Hz of spontaneous beating. (e) Typical waveform of FP recordings from spontaneous beating hES-CMs (single or several cells) on the single micro electrodes. Gray and red

lines indicate raw and smoothing at 200 point (20 ms), respectively.

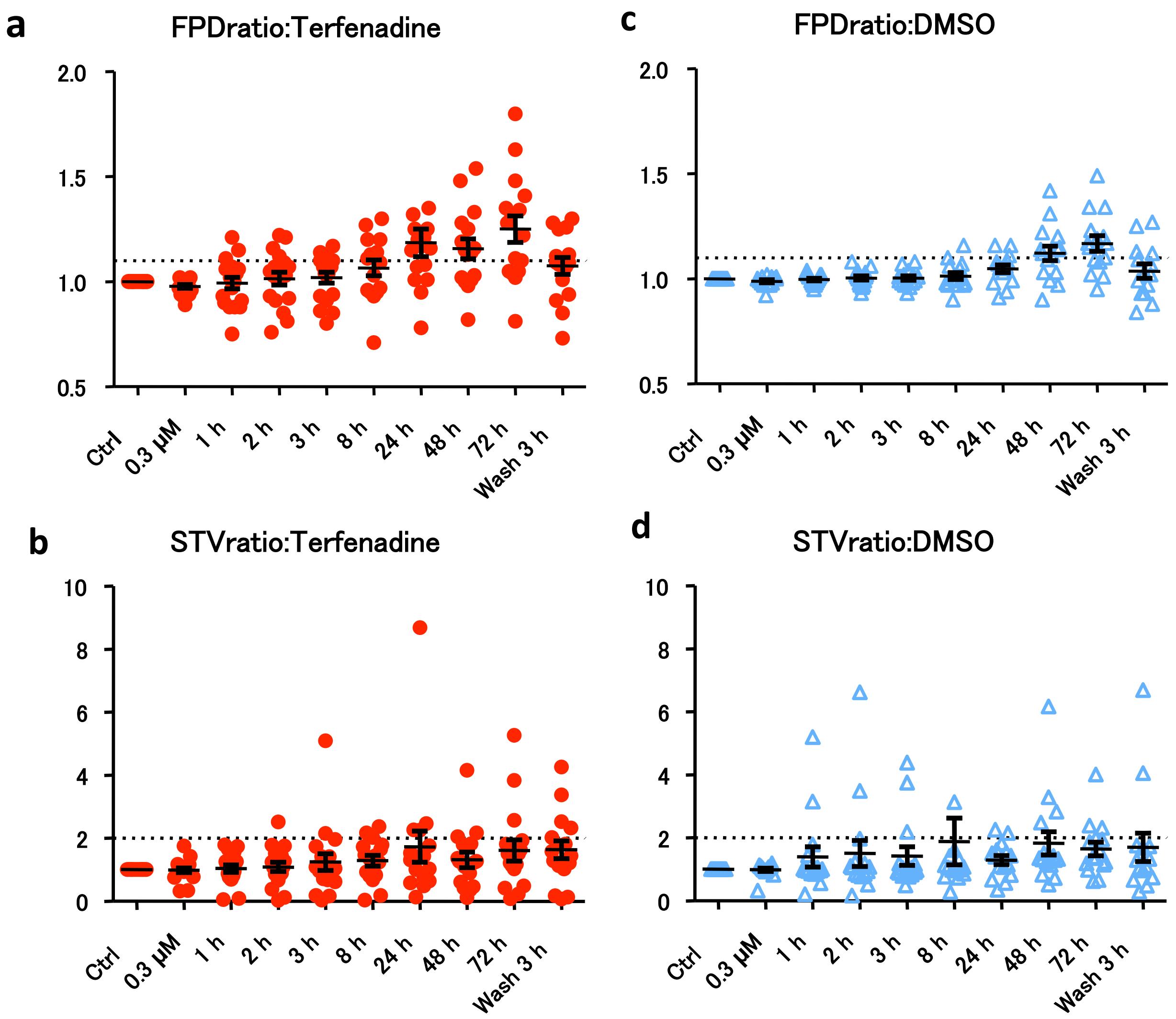

Supplementary Figure 5. Long-term observation of 0.3 μM terfenadine treated hCM clusters' FPD and STV. a, b, time course of FPD increase and STV_{FPD} increase of hCM clusters under 0.3 μM terfenadine treatment, respectively. **c, d,** time course of FPD increase and STV_{FPD} increase of hCM clusters under 0.03% DMSO in medium (control), respectively.

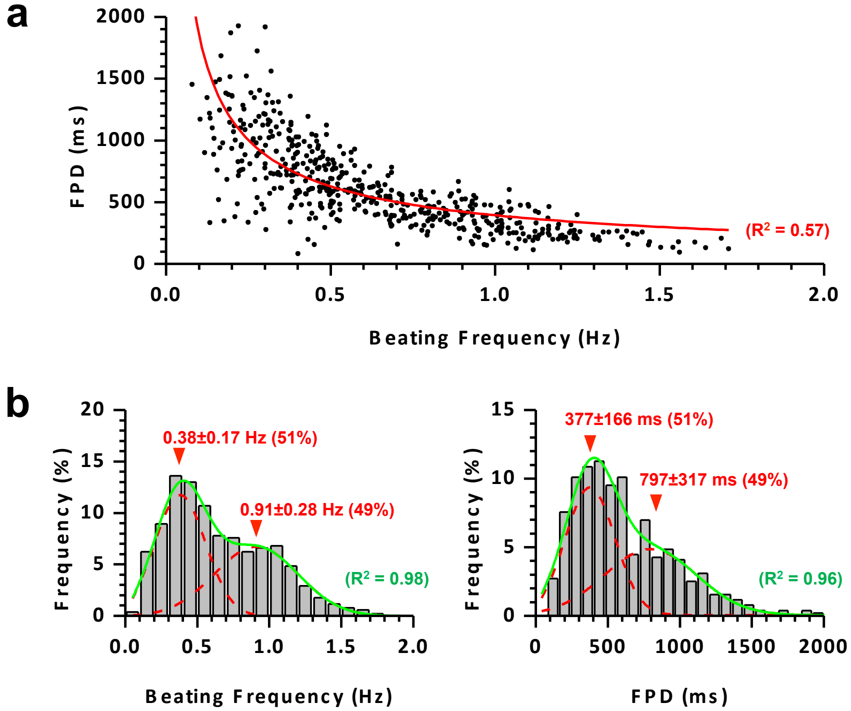

Supplementary Figure 6. Variety of beating properties on hES-CMs clusters. a, Relationship between BF and FPD on hES-CM clusters (Cellartis). n = 515 clusters. Red lines indicates the following allometric power fitting, $FPD = FPD_{1Hz} \times BF^{a}$, where FPD_{1Hz} (corrected with1 Hz, i.e. cFPD) are 394 ms, a are -0.67 (R^2 = 0.57). **b**, Histograms of beating frequency (left graph) and field potential duration (right graph) on hES-CMs clusters. Green lines indicate double Gaussian fitting curves.
